# The Preoperative Maximum Standardized Uptake Value Measured by 18F-FDG PET/CT as an Independent Prognostic Factor of Overall Survival in Endometrial Cancer Patients

**DOI:** 10.1155/2014/234813

**Published:** 2014-01-20

**Authors:** Malgorzata Walentowicz-Sadlecka, Bogdan Malkowski, Pawel Walentowicz, Pawel Sadlecki, Andrzej Marszalek, Tomasz Pietrzak, Marek Grabiec

**Affiliations:** ^1^Department of Obstetrics and Gynecology, The Ludwik Rydygier Collegium Medicum in Bydgoszcz, Nicolaus Copernicus University of Torun, Ujejskiego 75, 85-168 Bydgoszcz, Poland; ^2^Department of Nuclear Medicine, Lukaszczyk Oncology Center of Bydgoszcz, Poland; ^3^Department of Clinical Pathology, The Ludwik Rydygier Collegium Medicum in Bydgoszcz, Nicolaus Copernicus University of Torun, Poland

## Abstract

*Purpose*. The aim of this study was to determine if the preoperative maximum standardized uptake value (SUVmax) measured by 18F-FDG PET/CT in the primary tumor has prognostic value in the group of patients with endometrial cancer. *Patients, Materials, and Methods*. A total of one hundred one consecutive endometrial cancer patients, age range 40–82 years (mean 62 years) and FIGO I–IV stage, who underwent 18-FDG-PET/CT within two weeks prior radical surgery, were enrolled to the study. The maximum SUV was measured and compared with the clinicopathologic features of surgical specimens. The relationship between SUVmax and overall survival was analyzed. *Results*. The mean preoperative SUVmax was 14.34; range (3.90–33.80) and was significantly lower for FIGO I than for higher stages (*P* = 0.0012), as well as for grade 1 than for grade 2 and 3 (*P* = 0.018), deep myometrial invasion (*P* = 0.0016) and for high risk group (*P* = 0.0004). The analysis of survival ROC curve revealed SUVmax cut-off value of 17.7 to predict high risk of recurrence. Endometrial cancer patients with SUVmax higher than 17.7 characterized by lower overall survival. *Conclusion*. The preoperative SUVmax measured by 18F-FDG PET/CT is considered as an important indicator reflecting tumor aggressiveness which may predict poor prognosis. High value of SUVmax would be useful for making noninvasive diagnoses and deciding the appropriate therapeutic strategy for patients with endometrial cancer.

## 1. Introduction

Among all the cancers of female reproductive system, endometrial cancer became the most common in women in Europe and United States [1]. There were 5125 new cases diagnosed in 2010 in Poland, which makes endometrial cancer the third most common, after breast and lung cancer [2].

Natural course of endometrial cancer is slow and the disease is characterized by rather good prognosis [3]. However, for patients with advanced or recurrent disease, or for those who wish to preserve their fertility, limited treatment options are available. There is a group of patients with a poor prognosis, who will benefit from more aggressive treatment. This group will need adjuvant chemo- or radiotherapy. It is of great interest to learn more about the important risk factors predictive of recurrence and death [4]. The recognized, so far, poor prognostic factors for endometrial cancer are advanced FIGO stage, a nonendometrioid histological subtype, high grade (G3), deep invasion of myometrium (>50%), presence of lymph node metastasis, cervical involvement, and lymphovascular space invasion (LVSI) [5]. All risk factors mentioned above are identified after extensive surgical procedure. Considering the excellent prognosis of endometrial cancer, it seems to be of great importance to find the subgroup of patients with the good prognosis, who would not need comprehensive surgical staging and further treatment. It is especially important for older patients who suffer from severe concomitant diseases with high risk of complications during and after surgery [6]. It is also very important for young patients, who wish to preserve their fertility.

The new approaches for preoperative assessment of endometrial cancer are crucial to establish prognosis and plan the treatment [6]. The value of MRI and CT for preoperative staging is accepted but the role of assessment of deep myometrial invasion remains controversial [7]. None of the modalities can yet replace surgical staging. However, they all contribute to important knowledge and are, furthermore, able to upstage low-risk patients who would not have been recommended lymph node resection based on histology and grade alone [8]. Moreover, besides the difficulties with accurate preoperative evaluation of tumor extent, there are also difficulties with proper assessment of tumor grade and agressiveness [9]. The accuracy of preoperative and intraoperative assessment of histological grade was found to be around 90% only [10].

Whole-body PET/CT has excellent diagnostic tool for the overall assessment of distant metastases in patients with breast cancer [11]. Imaging with 18F-FDG PET/CT is a noninvasive diagnostic method that helps in predicting tumor features. Assessment of maximum standardized uptake valve (SUVmax) measured by 18F-FDG PET/CT is now recognized as a semi-quantitative parameter unique to PET, which is associated with tumor aggressiveness in numerous malignancies [12]. High SUVmax in primary tumors has been reported to correlate with higher proliferation in cancer cells due to increased rate of glucose uptake by tumor cells [13].

There are only few studies concerning relationship between SUVmax and endometrial cancer patients [8]. Therefore the aim of our study was to evaluate SUVmax measured by 18F-FDG PET/CT in relation to clinical outcome and overall survival.

## 2. Materials and Methods

### 2.1. Patients

One hundred and one consecutive endometrial cancer patients were enrolled to our prospective study between January 2007 and August 2008. All patients underwent total abdominal hysterectomy, with bilateral salpingoophorectomy and pelvic lymph node dissection performed by experienced gynecological oncologists at Department of Oncologic Gynecology of Ludwik Rydygier Collegium Medicum in Bydgoszcz, Nicolaus Copernicus University. All patients underwent 18F-FDG PET/CT within two weeks before surgery. Clinical stage was assessed based on the surgical specimens evaluation performed by two independent experienced pathologists according to International Federation of Gynecology and Obstetrics (FIGO) 2009 system. The study group included 29 patients with stage IA, 48 with stage IB, 13 with II, 11 women with stage III/IV (Table 1). Histological grade was assessed according to WHO classification (Table 1). Endometrioid subtype (Bokham type I) was diagnosed in 91 patients, nonendometrioid histological subtype (Bokham type II) in 10 women. The mean age at diagnosis was 62 years (range 40–82). Baseline characteristics of the study participants are enclosed in Table 1. According to risk factors all patients were divided into three groups: low risk—FIGO IA, G1 or G2, Bokhman type I (endometrioid); intermediate risk—IA G3, IB G1 or G2, Bokhman type I (endometrioid); high risk—all patients in type II (nonendometrioid), IB G3, FIGO II and higher. Patients from low-risk group did not receive any further treatment after surgery, women from intermediate risk group received brachytherapy (VBT) 5 weeks after surgery, and patients from high risk group underwent teleradiotherapy and VBT. Adjuvant chemotherapy was administered to ten patients with nonendometrioid histopathological subtype (chemotherapy consisted of carboplatin and paclitaxel).

In all cases overall survival was determined (in months). Only case with proven death related to cancer were analyzed. The follow-up time was 53–71 months (mean 61 months).

The Ethical Committee at the Ludwik Rydygier Collegium Medicum, Nicolaus Copernicus University of Torun approved this study protocol (decision No. KB 332/2007). All participants have provided the informed consent.

### 2.2. PET/CT Technique

Within two weeks prior to surgery all women underwent 18F-FDG PET/CT in the Department of Nuclear Medicine, Prof F. Lukaszczyk Oncology Center in Bydgoszcz. Patients were fasted min 6 hours before examination. In all patients the glucose serum concentration was measured just before planned FDG injection. Patients with the level of glucose higher than 150 mg/dl were excluded from the examination. Patients drank half a liter of water two hours before images acquisition, in order to facilitate urinary excretion. 18F-FDG (5–7 MBq/kg body weight) was administered intravenously. After 60 minutes a body scan was performed using combined PET/CT scanner. The PET and CT scans covered area from the base of the skull to the 1/3 upper thighs. Images were obtained using a PET/CT scanner (Biograph 6 and Biograph 16; Siemens, Germany). PET images were scatter-corrected and reconstructed with the use of an ordered-subset expectation maximization iterative reconstruction algorithm and a postreconstrution Gaussian filter (3 mm, full-width at half-maximum). The images were evaluated by two experience nuclear medicine physicians blinded to the clinical data of the patients'. The SUVs for FDG were calculated for the region of interested (ROI) using the standard formula. The maximum SUVmax was obtained from the image, which have had the highest SUVmax. The SUVmax was determined in all cases from the primary tumor within the uterus.

### 2.3. Statistical Analyses

All statistical analysis were performed using PQStat version 1.4.4.126. The statistical significance of SUV differences in relation to FIGO stage, grading (G) was assessed by the use of Kruskal-Wallis and Jonckheere-Terpstra test, the rest (lymph node status, depth of myometrial invasion, Bokhman type, and death before 60 months) with the use of *U* Mann-Whitney test.

Receiver operating test characteristics (ROC) curves were generated for SUVmax to determine the cutoff values for predicting survival in relation to risk groups, which yielded optimal sensitivity and specificity. Based on SUVmax cut-off values, patients were divided into two groups and Kaplan-Meier analysis was performed. With the use of the *F*. Cox's test and log-rank, the survival curves were analyzed. Moreover, multivariate analysis using Cox's proportional hazards models was performed to asses correlation between SUVmax and overall survival. The variables entered in the univariate analysis were FIGO stage, grading, Bokham's subtype, lymph node status, and depth of myometrial invasion. The variables entered in the multivariate analysis were FIGO stage, grading, Bokham's subtype, and depth of myometrial invasion. *P* < 0.05 was considered to be statistically significant.

## 3. Results

During the study period one hundred one consecutive endometrial cancer patients underwent FDG/PET CT scan. The mean preoperative SUVmax was 14.34; range (3.90–33.80). The mean SUVmax in relation to pathological parameters is shown in Table 2.

SUVmax was significantly lower for FIGO IA than for higher stages (*P* = 0.0012; Table 2). Increasing values of SUVmax were observed throughout FIGO stages. The mean SUVmax was significantly lower for grade 1, than for grade 2 and 3 (*P* = 0.018; Table 2). For myometrial invasion up to 50% the mean SUVmax was 12.16 and was statistically significantly lower comparing to cases with invasion ≥50% (SUVmax 15.59; *P* = 0.0016). Patients who died within 60 months of observation had significantly higher SUVmax comparing with women, who survived more than 5 years (17.14 versus 13.47, *P* = 0.0055). There were no significant differences in SUVmax according to lymph node status (Table 2).

Moreover we observed statistically significant correlation between SUVmax values and the risk group of recurrence. Patients from the low risk group had significantly lower SUVmax values, comparing to that from the intermediate and the high risk (*P* = 0.0004; Table 2).

Statistically significant correlations were found for SUVmax, FIGO stage, lymph node metastases, nonendometrial type II, and myometrial invasion ≥50% variables in the univariate Cox's regression for overall survival (OS) (Table 3). Grading was not found a significant variable for OS in the univariate analysis. Multivariate analysis using Cox's proportional hazard model according to risk groups showed statistically significant role of SUVmax, FIGO stage and deep myometrial invasion (Table 4). For SUVmax hazard ratio (HR) was >1 in univariate and multivariate analysis.

**Figure 1:**
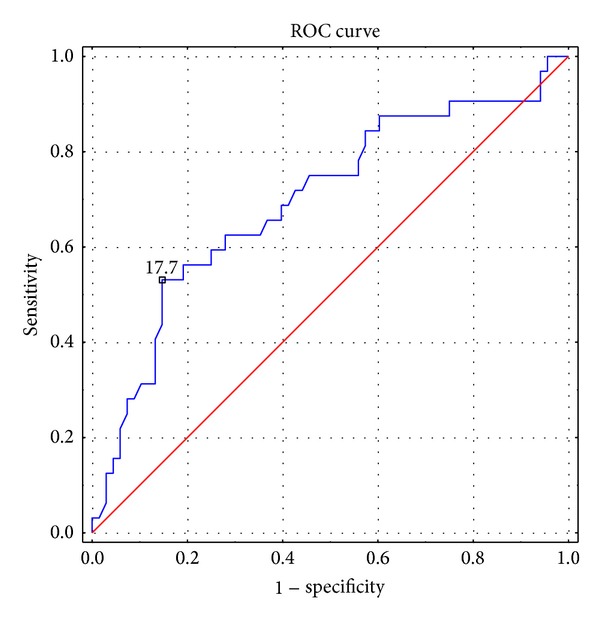
ROC curve for SUVmax to predict high-risk group.

To predict survival ROC curve analysis was used to determine SUVmax cut-off value (Figure 1).

The analysis revealed SUVmax cut-off value of 17.7 to predict high risk (AUC = 0.697 (SE = 0.059); CI −95% to 95% = 0.582–0.813; sensitivity 53.1%, specificity 85.3%). There were 69 patients with SUVmax lower than 17.7 and 32 women with SUVmax ≥17.7.

**Figure 2:**
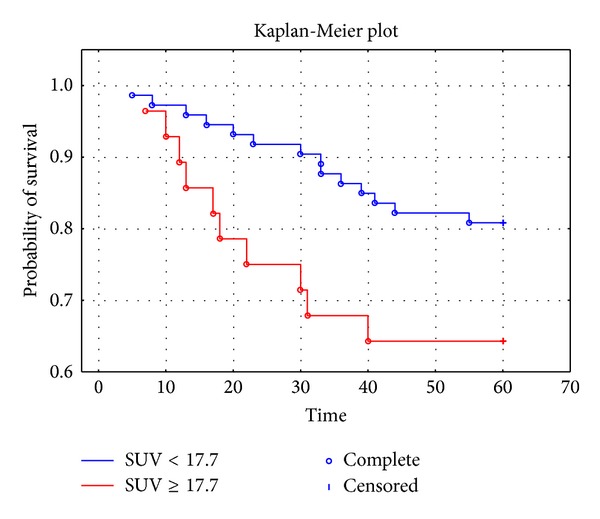
Kaplan-Meier curves for overall survival rates of patients categorized to SUVmax level of 17.7; *P* = 0.0366. Time in months.

According to the established cutoff value, patients were divided into two subgroups and Kaplan-Meier analysis was performed (Figure 2). There were significant differences observed. Patients with high SUVmax values (≥17.7) had overall survival rate shorter than those with lower SUVmax.

## 4. Discussion

The most important risk factors of overall survival and disease-free survival in patients with endometrial cancer include surgical stage, histology, depth of myometrial invasion, cervical involvement, lymph-node metastasis, and lymphovascular space invasion [14]. However, these parameters are not sufficient to predict individual prognosis in the beginning of treatment, as they can only be identified by surgical staging. Although surgical pathologic staging is now the standard of care for endometrial carcinoma; however, the role of pelvic and paraaortic lymphadenectomy for all patients remains controversial. Several reports asserted that patients with low-risk endometrial carcinoma can be treated with hysterectomy only [15]. Therefore, a noninvasive diagnostic method for preoperative risk stratification of survival and recurrence would be very useful. The presence of abnormal FDG uptake on 18F-FDG PET/CT images has been widely accepted as a criterion for differentiation between benign and malignant disease [6]. FDG uptake is mainly evaluated by calculation of the SUV [16–18]. A high SUVmax is not only correlated with tumor proliferation, but also associated with other signs of aggressive tumor behavior, including lymph node involvement and metastasis risk [19]. There are only few studies concerning the correlation between primary tumor assessment by PET/CT and clinical outcome of endometrial cancer. Therefore, the aim of our study was to evaluate preoperative metabolic activity in the primary tumor expressed by SUVmax and measured by 18F-FDG PET/CT and its relation to clinical outcome and overall survival. We were able to confirm that SUVmax of the primary endometrial cancer tumor correlated with FIGO stage, deep myometrial invasion and histological grade. Higher SUVmax values were observed in more advanced FIGO stage, deeper myometrial invasion, and higher histological grade. Similarly to our findings, Nakamura et al. found that SUVmax was associated with FIGO stage, grading, and myometrial invasion; moreover, statistical significance in lymph node status was not proven, similar to our findings [20, 21]. Lee et al. reported that preoperative SUVmax for the primary tumor in 60 patients with endometrial cancer (FIGO stage I to III) was significantly associated in multivariate analysis with FIGO stage, histological grade, myometrial invasion, LVSI, and maximum tumor size [22]. Torizuka et al. demonstrated that primary tumor SUVmax in 22 patients with endometrial cancer (FIGO stage I) increased with myometrial invasion and maximum tumor size (SUV cutoff was 12.0). [23].

There are only a few studies that investigated the value of preoperative SUVmax for predicting recurrence risk in patients with endometrial cancer after radical surgery. Kitajima et al., in multivariate analysis showed that SUVmax was significantly associated with recurrence (*P* = 0.045, HR 1.11, 95% CI 1.0028–1.231). They set SUVmax cut-off value of 12.7 to show differences in disease-free survival rate [24]. Probably the reason why lower cut-off value was used in their study was a little different patients characteristics (the mean value of the SUVmax for all patients was 10.6) and the analysis concerned disease-free survival, not overall survival [24].

Nakamura et al. demonstrated that not only the DFS but also OS rates of patients showing a high SUVmax of the primary tumor were significantly lower than those of patients showing a low SUVmax (*P* = 0.049 and *P* = 0.039, resp.). They showed that the SUVmax of the primary tumor was an independent prognostic factor for an overall survival in a multivariate analysis (*P* = 0.025) [21]. To show the significance in survival rates between patients, authors set the SUVmax cut-off value of 16.5. In their next report, based on ROC curve the optimal cut-off value for predicting survival was SUVmax 18.41 [25].

The three studies mentioned above proved the correlation between preoperative SUVmax for the primary tumor on FDG PET/CT and other known prognostic factors, as well as being one of the most powerful and significant predictive factor of endometrial cancer prognosis.

In our study we examined the SUVmax of the endometrial cancer and its correlation with clinical characteristics in patients with primary endometrial cancer. We confirmed that SUVmax of the primary tumor in our group of patients had a significant association with overall survival. We found that preoperative measurement of the SUVmax of primary tumor was directly associated with the prognosis of endometrial cancer. The mean SUVmax in the group of women who survived less than 60 months was significantly higher compared to ones who survived more than 5 years (17.14 versus 13.46, *P* = 0.0055). Based on Cox's analysis we confirmed that SUVmax was an independent prognostic factor of overall survival in the endometrial cancer group. With the use of ROC curve we set the optimal cut-off value to predict high risk as 17.7. Kaplan-Meier analysis showed significantly important inferences in survival rates between both groups of patients. Patients with SUVmax ≥17.7 were characterized with worse prognosis.

It is really important to have a possibility to use noninvasive techniques, such as 18F FDG PET/CT in selected group of endometrial cancer patients, especially older women, who are at high risk of severe postsurgical complications. With the use SUVmax assessed at FDG PET/CT, we are more likely to select patients that are in high risk group, who would benefit from aggressive surgery and further treatment taking a risk of complications. SUVmax reflects the glucose metabolism and it is related to oncogene activation, hypoxia, and angiogenesis, and, finally, it is connected to tumor aggressiveness [26]. In our study we were able to confirm the relationship between SUVmax and poor prognosis in endometrial cancer patients. The measurement of SUVmax of the primary tumor did not influence the further treatment. All patients were treated according to the histopathological findings. The main limitation of the study was a relatively small group of patients, but in spite of that fact we were able to confirm the association of SUVmax and overall survival. In conclusion, our results provide evidence that preoperative staging with SUVmax value of the primary tumor could be used to select patients with the poor prognosis and choose an adequate surgical approach for them. Further prospective studies are needed to confirm beneficial effects of SUVmax on the treatment decisions and outcome of women diagnosed of endometrial cancer.

## 5. Conclusion

The preoperative SUVmax measured by 18F-FDG PET/CT is considered a very informative index reflecting tumor aggressiveness which may predict poor prognosis. High value of SUVmax would be useful for making noninvasive diagnoses and deciding the appropriate therapeutic strategy for patients with endometrial cancer.

## Figures and Tables

**Table 1:** Baseline characteristics of study participants.

Baseline characteristics	All patients	
Age at diagnosis, years	Mean, 62; range, 40–82	
	Number of patients	(%)
FIGO stage		
IA	29	28.71
IB	48	47.52
II	13	12.87
IIIA	3	2.97
IIIB	3	2.97
IIIC	4	3.96
IVA	1	0.99
Lymph node metastasis		
0	97	96.04
1	4	3.96
Distant metastasis		
0	99	98.02
1	2	1.98
Myometrial invasion ≥50%		
0	38	37.62
1	63	62.38
Grading		
1	13	12.87
2	74	73.27
3	14	13.86
Histopathological subtype (Bokham type)		
1	91	90.10
2	10	9.90
Risk group		
Low	29	28.71
Intermediate	39	38.61
High	33	32.67
Death (before 60 months)		
0	77	76.24
1	24	23.76

**Table 2:** The mean SUVmax in relation to pathological parameters.

	Mean	Standard deviation (SD)	Lower quartile (Q1)	Median (Me)	Upper quartile (Q3)	*P*	
FIGO stage							
IA	11.45	6.14	7.60	9.70	13.60	a	0.0012
IB	14.61	4.37	11.10	15.20	17.35	b	
II	17.09	5.75	16.40	19.50	20.40	b	
III + IV	17.55	7.90	14.00	18.10	19.20	b	
Lymph nodes metastasis							
Positive	17.15	12.12	9.40	15.00	24.90	0.8191
Negative	14.20	5.57	9.30	14.15	18.15	
Depth of myometrial invasion							
≥50%	15.59	5.02	12.20	15.60	19.00	0.0016
<50%	12.16	6.65	7.40	10.10	16.10	
Grading							
1	11.25	4.70	9.10	9.20	15.20	a	0.0180
2	14.45	5.94	9.40	14.40	18.20	ab	
3	16.66	5.46	12.10	16.20	20.20	b	
Histopathological subtype (Bokham type)							
1	14.12	5.82	9.20	14.30	18.10	0.3813
2	16.31	6.12	12.20	14.20	20.20	
Death (before 60 months)							
Yes	17.14	5.09	14.20	17.10	19.35	0.0055
No	13.47	5.83	9.10	12.80	17.40	
Risk groups							
Low	11,45	6,14	7,60	9,70	13,60	0,0004
Intermediate	14,24	4,20	10,40	15,20	17,10	
High	16,92	6,28	12,90	17,80	20,25	

**Table 3:** Univariate Cox's regression for overall survival.

	Beta	Error deviation	*P*	Hazard ratio	−95% confidence interval	+95% confidence interval
SUVmax	0,093291	0,032927	**0,004607**	1,097782	1,029174	1,170963
FIGO (III + IV)	−1,05204	0,219490	**0,000002**	0,121957	0,051588	0,288315
Grading G2 + G3	−0,034713	0,308660	0,910457	0,932930	0,278218	3,128335
Nonendometrioid type II	−0,687563	0,236101	**0,003589**	0,252808	0,100196	0,637867
Positive lymph nodes	−2,56541	0,229951	**0,000120**	0,005912	0,002400	0,014561
Myometrial invasion ≥50%	−0,562819	0,275139	**0,040798**	0,324446	0,110343	0,953979

**Table 4:** Multivariate Cox's regression for overall survival.

	Beta	Error deviation	*P*	Hazard ratio	−95% confidence interval	+95% confidence interval
SUVmax	0,06488	0,032866	**0,048369**	1,067032	1,000465	1,138029
FIGO (III + IV)	−1,34704	0,309160	**0,000013**	0,067605	0,020122	0,227140
Grading G2 + G3	0,25922	0,318119	0,415155	1,679409	0,482603	5,844172
Non-endometrioid type II	−0,04632	0,279662	0,868451	0,911523	0,304558	2,728131
Myometrial invasion ≥50%	−0,92271	0,312223	**0,003124**	0,157958	0,046453	0,537122
